# Pond Acoustic Sampling Scheme: A draft protocol for rapid acoustic data collection in small waterbodies

**DOI:** 10.1002/ece3.7585

**Published:** 2021-05-01

**Authors:** Carlos Abrahams, Camille Desjonquères, Jack Greenhalgh

**Affiliations:** ^1^ Baker Consultants Ltd Matlock UK; ^2^ Nottingham Trent University Nottingham UK; ^3^ Behavioral and Molecular Ecology Group Department of Biological Sciences University of Wisconsin‐Milwaukee Milwaukee WI USA; ^4^ School of Biological Sciences University of Bristol Bristol UK

**Keywords:** acoustic monitoring, bioacoustics, ecoacoustics, pond, rapid assessment methods, soundscape, survey

## Abstract

1. Freshwater conservation is vital to the maintenance of global biodiversity. Ponds are a critical, yet often under‐recognized, part of this, contributing to overall ecosystem functioning and diversity. They provide habitats for a range of aquatic, terrestrial, and amphibious life, often including rare and declining species.
2. Effective, rapid, and accessible survey methods are needed to enable evidence‐based conservation action, but freshwater taxa are often viewed as “difficult”—and few specialist surveyors are available. Datasets on ponds are therefore limited in their spatiotemporal coverage.
3. With the advent of new recording technologies, acoustic survey methods are becoming increasingly available to researchers, citizen scientists, and conservation practitioners. They can be an effective and noninvasive approach for gathering data on target species, assemblages, and environmental variables. However, freshwater applications are lagging behind those in terrestrial and marine spheres, and as an emergent method, research studies have employed a multitude of different sampling protocols.
4. We propose the Pond Acoustic Sampling Scheme (PASS), a simple protocol to allow a standardized minimal sample to be collected rapidly from small waterbodies, alongside environmental and methodological metadata. This sampling scheme can be incorporated into a variety of survey designs and is intended to allow access to a wide range of participants, without requiring complicated or prohibitively expensive equipment.
5. Adoption of this sampling protocol would enable consistent sound recordings to be gathered by researchers and conservation organizations, and allow the development of landscape‐scale surveys, data sharing, and collaboration within an expanding freshwater ecoacoustic community—rather than individual approaches that produce incompatible datasets. The compilation of standardized data would improve the prospects for effective research into the soundscapes of small waterbodies and aid freshwater conservation efforts.

## Graphical abstract

New acoustic survey methods are becoming increasingly available to researchers, citizen scientists, and conservation practitioners. We propose a Pond Acoustic Sampling Scheme (PASS), to allow standardized individual audio samples to be collected rapidly from small waterbodies, alongside environmental and survey metadata. This will allow a wide range of participants, without complicated/expensive equipment, to contribute to collaborative data collection projects, and enable the development of the consistent datasets often missing from ecoacoustics research.

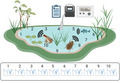

## 1 INTRODUCTION

### 1.1 Pond conservation

Freshwater biodiversity is globally threatened by overexploitation, pollution, hydrological modification, habitat destruction, and invasive species (Cantonati et al., 2020; Dudgeon et al., 2006). These impacts, exacerbated by the interconnected nature of freshwater ecosystems, have resulted in population declines and species distribution changes, with consequences for a range of ecosystem services.

Even though ponds (small waterbodies <2 ha in area) can be relatively abundant in many landscapes and provide critical habitats for diverse floral and faunal communities, they have been under‐recognized and neglected compared with larger freshwater habitats (Biggs et al., 2005; Bolpagni et al., 2019; Wood et al., 2003). Ponds are physically and biologically heterogeneous habitats, which offer migration stepping stones and breeding sites for aquatic, amphibious, and terrestrial species, and can support regional metapopulations and a high proportion of rare species (De Meester et al., 2005; Williams et al., 2004). Due to this diversity and function, pond ecosystems contribute significantly to freshwater (and terrestrial) biodiversity across the globe (Indermuehle et al., 2010; Williams et al., 2004). Despite their value, ponds are not covered by legal protection and policy in the same way that larger lakes and rivers are (Bolpagni et al., 2019; Hill et al., 2018), limiting options for their protection and enhancement.

In terms of scientific research, ponds also offer good model systems for surveys or hypothesis testing through experimental manipulation, providing potential for studies in ecology, evolutionary biology, and conservation biology (De Meester et al., 2005). The majority of recent publications on ponds have covered the interactions between environmental factors and species spatial patterns (focusing on zoobenthos), and have had a distinct applied research character, with increasing interest in methodological studies (Bolpagni et al., 2019).

### 1.2 Pond survey

Effective and accessible survey methods are needed to enable evidence‐based conservation action. However, established standard methods for the assessment of ponds are rare. The Predictive SYstem for Multimetrics (PSYM) was developed in the late 1990s, followed later by PLOCH and IBEM methods (Biggs et al., 2000; Indermuehle et al., 2010; Oertli et al., 2005), to allow assessment of the biological quality of ponds using aquatic plants and macroinvertebrates. However, these methods are all limited in their geographic applicability, the types of ponds to which they can be applied, the time and resource requirements for implementation, and the considerable amount of identification expertise needed to get reliable results (Biggs et al., 2000; Harper et al., 2019; Indermuehle et al., 2010; Labat, 2017; Oertli et al., 2005; Pond Conservation, 2010). As a result, ponds have often been neglected in limnological studies, and there is limited scientific knowledge of pond ecology (Mainstone et al., 2018; Oertli et al., 2005). The ecological basis for pond management is therefore poorly established, with practical conservation efforts often led by management “myths” rather than solid evidence (Biggs et al., 2005).

To enable accessible and efficient pond survey and monitoring, the need for a “Rapid Assessment Method” for ponds has been recognized (Labat, 2017; Menetrey et al., 2005; Pond Conservation, 2010; Sueur, Pavoine, et al., 2008). A Rapid Assessment Method is a standardized procedure that allows efficient generation of an index score, representing the ecological status or ecosystem function of a particular site, and summarizing key components of habitat integrity (hydrological, physical, chemical, and biological; Dorney et al., 2018; Mainstone et al., 2018). Developing such an approach for ponds would have value for researchers and citizen scientists, meeting a clear requirement for (i) improved collation and sharing of harmonized data, (ii) the integration of biological, physical, and chemical parameters, and (iii) increased geographical coverage of information on pond quality and biodiversity (Cantonati et al., 2020; Heino et al., 2020).

Although existing survey approaches, using invertebrate and macrophyte data, have significant value (Biggs et al., 2005; Bolpagni et al., 2019), there is an obvious need for expansion of widely applicable assessment tools that can develop coherent and transferable field data and metrics. Developments in technology are currently enabling such new approaches (August et al., 2015). For example, the use of environmental DNA and metabarcoding allows the identification of single species or assemblages from a simple water sample (Harper et al., 2019; Lim et al., 2016). The use of underwater sound recordings could offer the potential to assess pond habitats with minimally intrusive and easily employed field visits, allowing the identification of taxa present or calculation of overall metrics of environmental quality (Sueur, Pavoine, et al., 2008). Here, we propose the Pond Acoustic Sampling Scheme (PASS), a simple draft protocol to allow standardized minimal samples to be collected rapidly from small waterbodies.

### 1.3 Freshwater ecoacoustics

Many freshwater taxa produce sound—notably fish, arthropods, and amphibians (Desjonquères et al., 2020; Linke et al., 2018). In addition, environmental sounds are also created by water flows, wave action, and gaseous exchange in macrophytes and pond substrates (Linke et al., 2018). These natural sounds, alongside anthropogenic noise, can all be captured using underwater microphones (hydrophones) to provide data on pond ecosystems (Greenhalgh et al., 2020; Kuehne et al., 2013; Linke et al., 2018; van der Lee et al., 2020). The benefits of using acoustic recording, especially alongside traditional surveys, are well documented from scientific research in other habitats. In particular, the ability to produce a standardized, long‐duration, permanent dataset, which can be repeatedly analyzed, and subject to quality assurance checks, is a major advantage over standard field surveys (Desjonquères et al., 2020; Linke et al., 2018; Sugai, Silva, et al., 2019). The use of ecoacoustics in scientific research has therefore increased significantly over the last ten years—and studies in freshwaters are becoming more common (Greenhalgh et al., 2020). Acoustic surveys can clearly only capture sounds from soniferous taxa, and a further current disadvantage is that the knowledge of sounds produced by different freshwater species is highly limited (Rountree et al., 2020). In addition, the recent emergence of the field means that there are no agreed standards for sampling the soundscape of a given habitat, and guidance is also lacking on how recordings can best be used for effective biodiversity monitoring (Bradfer‐Lawrence et al., 2019; Sugai, Silva, et al., 2019).

A recent review of the freshwater bioacoustics literature (Greenhalgh et al., 2020) identified a bias toward single‐species studies of fish sounds (44% of studies), conducted in a laboratory setting (53%). Pond habitats were included in just 11% of studies, and aquatic arthropods were only represented in 26% of studies, despite their significant contributions to freshwater ecosystem function and soundscape composition. The soundscapes of temperate freshwater ponds were not investigated at all prior to the study by Desjonquères et al. (2015). Despite these current gaps in the research literature, ecoacoustic methods have revealed differences in the freshwater soundscapes over different types of sites and across environmental gradients (Desjonquères et al., 2018; Kuehne et al., 2013; van der Lee et al., 2020). In perhaps the largest‐scale study to date, Rountree et al. (2020) recorded the soundscape of 19 lakes, 17 ponds, 20 rivers, and 20 streams in New England (USA), capturing 7,000 sounds at 173 sampling locations. They found that freshwater habitats contain a diverse array of unidentified biological sounds and that anthropogenic noises (transport, boats, fishing) dominated the recorded soundscapes, imposing significantly on natural sounds.

Recent developments in acoustic sensors and automated processing methods now allow researchers to collect and process large datasets of recordings (Sethi et al., 2020; Sueur, Pavoine, et al., 2008). This ability is rapidly expanding the field of acoustic research in freshwaters, but the majority of studies to date have focused on temporal rather than spatial variability, targeting a limited number of waterbodies over long periods, with autonomous acoustic recorders (Desjonquères et al., 2015; Karaconstantis et al., 2020). There is, however, considerable benefit in focal recording by surveyors, with active listening in the field, as opposed to later playback and analysis. This approach allows for a deeper understanding of the diversity of sounds present and can prevent the misidentification of some anthropogenic and environmental sounds coming from biological sources (Rountree et al., 2020). Despite this benefit, very few studies have undertaken this approach. Rountree et al. (2020) conclude that researchers should attempt to increase the number of studies using real‐time sound monitoring in the field, with visual observations of the recorded soundscape, alongside other projects that focus on the collection of long‐term soundscape recordings.

### 1.4 Aims of the PASS

This paper does not set out to describe a survey method. Similar to a five‐minute point count for birds (Bonthoux & Balent, 2012), or a three‐minute net sample for aquatic invertebrates (Hill et al., 2016; Williams et al., 2004), we simply suggest an approach to standardize the collection of a single audio sample recording the soundscape of a pond. This individual data capture can be employed within a wide variety of survey designs, based on the needs of the study, enabled by the multipurpose nature of the raw audio data. Sugai, Desjonquères, et al. (2019) identified three main challenges for the expansion of ecological acoustic research: nonstandardized monitoring procedures, time‐consuming acoustic analysis, and limitations on data curation and data sharing. This draft protocol is intended to address the first and last of these.

Despite the potential benefits of acoustic survey in freshwaters, there are currently no recognized standard field methods. We aim to support filling this gap at an early stage in practice development, by promoting coherent data gathering that will allow effective data sharing between surveyors and studies. While recognizing the potential disadvantages to defining set methods when the science is still developing, we believe that a standardized sampling protocol would have considerable benefits to the uptake of the ecoacoustics approach in freshwaters and the usability of the data collected.

We hence propose a simple protocol to allow standardized minimal samples to be collected from small waterbodies, producing a sound recording with associated environmental information and metadata. The protocol is intended to be accessible to a wide range of users, including researchers, consultants, conservation managers, and citizen scientists, without requiring complicated or expensive equipment. It is designed for use with a single handheld recorder and hydrophone, and for short site visits.

This sampling protocol should be built into a defined survey plan with additional guidance on spatial and temporal coverage, for example, to generate data across a range of sites for a regional survey, or to allow long‐term monitoring of ponds through repeated visits. The proposed sampling method is expected to yield useful data on pond soundscapes and lead to an improved understanding of how these relate to wider ecological function and site condition. Uptake of this method would allow consistent data to be gathered by a range of interested parties, allowing much‐needed data sharing and collaboration in this developing area (August et al., 2015; Linke, Gifford, et al., 2020). The recordings can also be used to document freshwater soundscapes for educational, artistic, or historical purposes (Barclay et al., 2020; Sugai & Llusia, 2019). We invite feedback from contributors to further develop good practice and demonstrate how this sampling protocol can be applied in full studies.

## 2 SAMPLING PROTOCOL

### 2.1 Recording the sound sample

**FIGURE 1:**
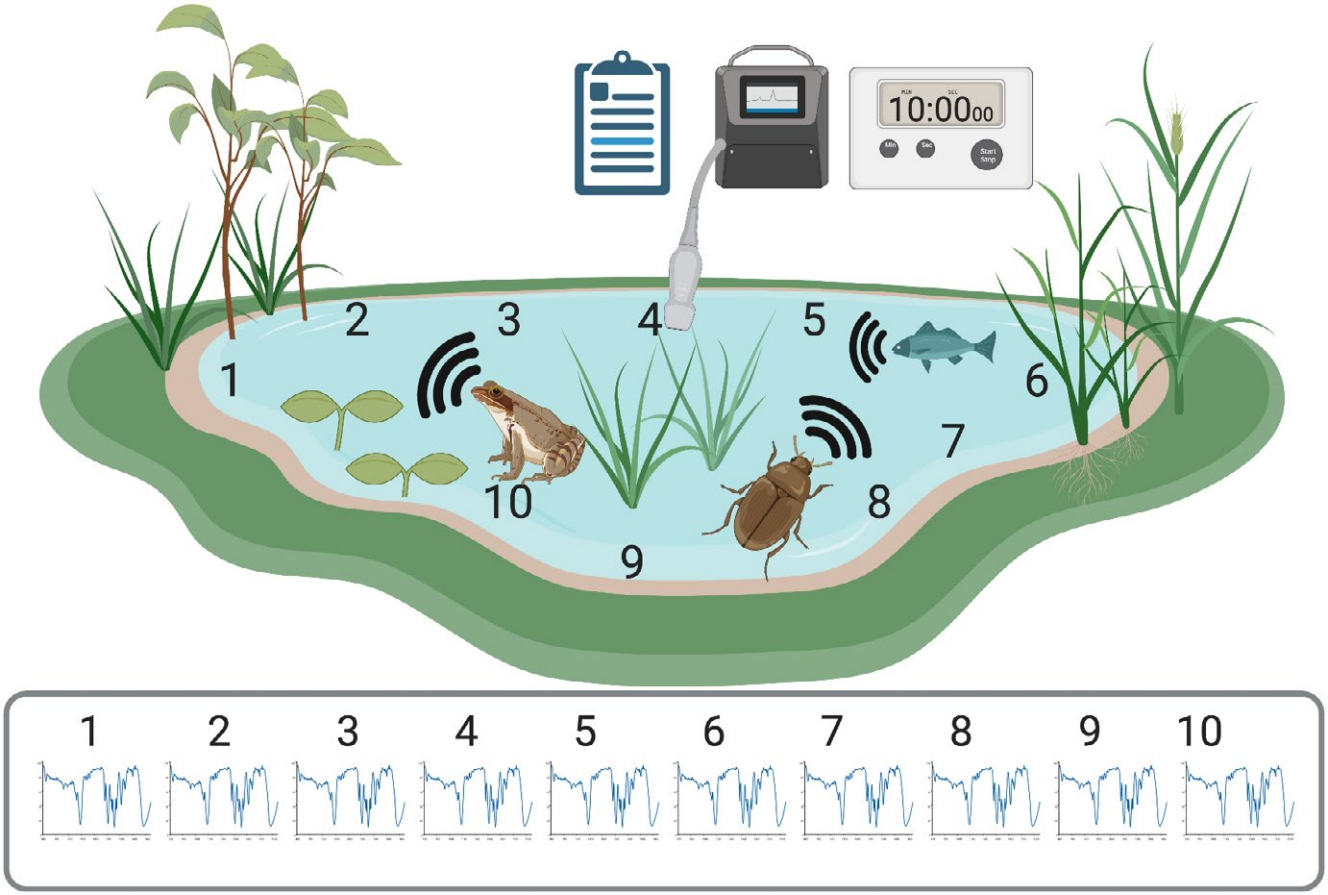
Pond Acoustic Sampling Scheme. Each sample consists of a 10‐min underwater sound recording from the pond, comprising 10 recordings, each of 1‐min duration, taken at different locations around the waterbody. Environmental parameters and survey metadata are systematically collected to accompany each sound sample

The sound recording collected for each sample is a 10‐min recording, saved as an uncompressed .WAV file. To represent potential variation across the waterbody, each 10‐min sample should be divided into ten 1‐min subsamples recorded in different mesohabitats around the edge of the pond (Figure 1). The 1‐min recording length has become common practice for ecoacoustic research, used in many studies (e.g., Bayne et al., 2017; Campos‐Cerqueira et al., 2020; Eldridge et al., 2018; Farina et al., 2011; Farina & Gage, 2017; Fuller et al., 2015; Gottesman et al., 2018; Pieretti et al., 2015; Wimmer et al., 2013), and has benefits over longer recording periods in terms of acoustic index accuracy, and computational requirements (Cifuentes et al., 2021). The 10‐min survey time is suggested as the minimal survey effort required for each sample and is partly pragmatic, based on keeping field visits to each pond of a reasonably short duration, and thereby enabling more sites to be visited in one field day. However, the review by Sugai, Silva, et al. (2019) of 460 published acoustics studies showed that 91% of those using discontinuous recording used sample lengths of 10 min or less. In addition, existing protocols of traditional surveys using auditory cues can offer guidance to determine recording lengths for acoustic monitoring. For long‐term monitoring of amphibian population trends, call surveys with 3–5 min lengths per hour have been shown to be adequate for most species (Dorcas et al., 2009; Shirose et al., 1997), whereas for birds, studies have often used lengths of 5–20 min (Bonthoux & Balent, 2012). Similar recording lengths have also been used for insects, for example, 3‐min recordings (Thompson et al., 2019). Critically, previous research has commonly found that acoustic diversity is better represented with a greater number of short‐duration samples than with fewer, longer‐duration samples (Bayne et al., 2017; Linke, Decker, et al., 2020; Sugai, Desjonquères, et al., 2019). This is particularly true if those visits are spread across times, days, and seasons (Browning et al., 2017). We therefore consider that 10 recordings of 1 min is a valid design choice, supported by a considerable body of research and established practice—and one that also allows efficient processing of the sound files by R software (Jorge et al., 2018).

When recording the sample, the hydrophone should be deployed at approximately 10 cm below the surface, and allowed to settle prior to starting the recording to allow any noise from air bubbles or vegetation movement to cease. The ten recording locations should be arrayed around the pond to sample the mesohabitats present, for example, marginal vegetation, submerged vegetation, and open water, in accordance with their relative area, and to capture the diversity of soniferous animals likely to be present (Aiken, 1991).

The sound file should be stored as a single 10‐min .WAV file to ensure that the recordings from a single sample remain together. This can either be achieved by using the recorder pause button between subsamples while in the field, or by recording 10 separate files and combining these together into one file after the field visit. The first approach may be easier, but less accurate in timing. The latter would allow files in excess of 60 s to be recorded and then cut accurately to length, before stitching them together, and hence would allow potential overlaps or inaccuracies in the length of subsamples to be avoided. Once recorded, files should be archived using a file naming protocol that includes a prefix (e.g., location and surveyor name), followed by date and time: PREFIX_YYYYMMDD_HHMMSS.wav. This convention follows the Wildlife Acoustics Song Meter system and is machine‐readable using seewave::songmeter in R (Sueur, Aubin, et al., 2008).

### 2.2 Recording equipment

**FIGURE 2:**
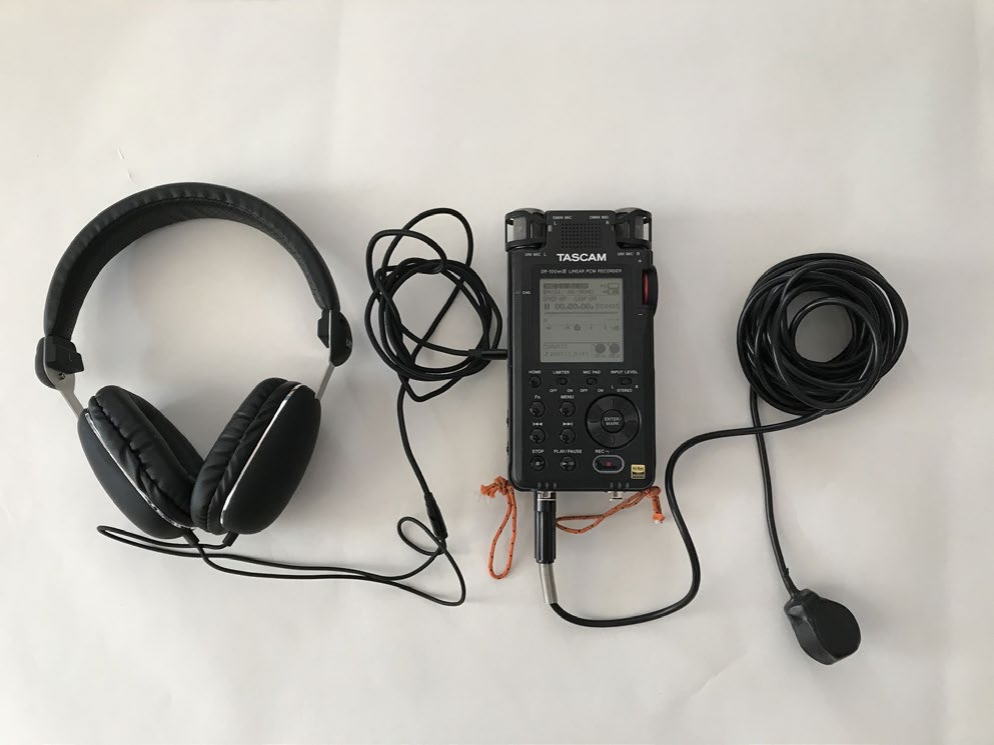
Typical recording equipment for PASS, consisting of headphones, recording unit, and cabled hydrophone

The 10‐min sound sample is recorded using a hydrophone and connected sound recording device (Figure 2). A range of manufacturers and models are available, and any of these can be used for this protocol (see Box 1 and Tables 1 and 2 for examples). The critical issue is to make sure that the equipment used is recorded in survey metadata, together with audio settings such as the use of frequency filters. Recorders should have low self‐noise, and the hydrophone should have a flat response across the range of audible frequencies.

**TABLE 1:** Hydrophones available for use in freshwater ecoacoustic surveys

Hydrophone model	Manufacturer	Cost (£)	Sensitivity (dB re: 1V/µPa)	Flat frequency response range	Compatible with
Standard/D series	Jez Riley French	50	N/A	N/A	Any device with a 3.5 mm or 1/4 microphone input
H2a	Aquarian Audio	148	−180	20 Hz to 4 kHz	Any device with a 3.5 mm microphone input
SQ26‐H1B	Cetacean Research Technology	N/A	−169	20 Hz to 45 kHz	Any device with a 3.5 mm microphone input
Pro	Dolphin Ear	320	N/A	1 Hz to 24 kHz	Any device with XLR connection
HTI‐96	High Tech, Inc.	N/A	−165 (with preamp)	2 Hz to 30 kHz	Any recorder

**TABLE 2:** Commonly used field recorders in freshwater ecoacoustic research

Recorder	Manufacturer	Cost (£)	Maximum battery life (hr)	Number of *SD* card slots	Bit depth	Maximum sample rate (kHz)	Maximum gain (dB)	Weather‐proof?	Programmable recording schedules?
H1n	Zoom	80	10	1	16, 24	96	39	No	No
H2n	Zoom	112	50	1	16, 24	96	39	No	No
DR‐100 MKIII	Tascam	250	12	1	16, 24	192	24	No	No
SM4 BAT FS	Wildlife Acoustics	743	230	2	16	500	12	Yes	Yes
AudioMoth (version 1.2.0)	Open Acoustic Devices	60	192	1	16	384	N/A	Yes	Yes

Manufacturers such as Zoom, Tascam, and Olympus produce a range of handheld field recorders that differ in the number of available channels, maximum gain settings, battery life, and price. However, relatively inexpensive and effective setups can be purchased that are well suited for short‐duration acoustic surveys.

A handheld Zoom recorder (e.g., models, H2, H4n, and H6) in combination with the H2a Aquarian Audio hydrophone is a popular equipment choice among some researchers (Decker et al., 2020; Karaconstantis et al., 2020; Linke, Gifford, et al., 2020). Rountree and Juanes (2020) used a Cetacean Research Technology SQ26‐H1B hydrophone and Zoom H1n recorder to describe the sounds produced by six piranha species in the Pacaya‐Samiria National Reserve, Peru. Other hydrophones used to record fish sounds in the field have included Cetacean Research Technology SQ26‐08 and C54XR, and the High Tech Inc. 96‐min (Rountree et al., 2018, 2020). Desjonquères et al. (2015) used Wildlife Acoustics SongMeters with RESON TC 4033 to record in ponds, while Gottesman et al. (2018) and Desjonquères et al. (2018) used a SongMeter with a HTI‐96 hydrophone for deployment in a swamp and secondary river channels, respectively. Other autonomous recorders such as the new AudioMoth 1.2 version with potential for a 3.5 mm jack input (https://www.openacousticdevices.info/audiomoth), or the Frontier Labs Bioacoustic Audio Recorder (https://frontierlabs.com.au/bioacoustics.html) are potential alternatives.

BOX 1 **Potential equipment setups for Pond Acoustic Sampling Scheme (and general freshwater acoustics work) varying in sensitivity and price**

Inexpensive handheld survey option: JRF standard hydrophone, with Zoom H2n recorder (total cost = £165)

Moderately priced survey option: Aquarian H2a hydrophone and Tascam DR‐100 recorder (total cost = £400)

Expensive survey option: Dolphin Ear Pro hydrophone with Zoom F8 recorder (multitrack) (total cost = £850)

Automated survey option: Aquarian H2a hydrophone, with AudioMoth recorder (version 1.2.0) (total cost = £208)

### 2.3 Audio settings

To ensure high‐quality sound data, recordings should be made with a sample rate of 44.1 or 48 kHz, and 16 or 24 bit depth. These recording parameters will ensure that the sound amplitude is recorded at high resolution, and enable recording of sounds up to 24 kHz, hence covering the range from low frequency fish sounds (Popper & Hawkins, 2019) to higher frequency invertebrate stridulations (Aiken, 1985). Lossless .WAV files should be used, rather than .MP3, to ensure that sound quality is not lost through file compression.

Recording volume (amplitude) is controlled by the gain setting on the recorder. The appropriate level is dependent on the equipment used and the sound levels in the waterbody, so needs to be set by the surveyor. It is normal in acoustic recording to set the peak amplitude to reach −6dB to prevent “clipping” and distortion of the noise files. Manufacturer recommendations should be referred to here, and some trial and error will be involved.

### 2.4 Metadata and environmental information

A standard data form is provided (PDF and CSV in Data S1) for recording environmental information about the waterbody, together with survey metadata. This has been designed for compatibility with the information collected for two existing survey methods in the UK: the Great Crested Newt Habitat Suitability Index (Oldham et al., 2000; https://www.arguk.org/info‐advice/advice‐notes/9‐great‐crested‐newt‐habitat‐suitability‐index‐arg‐advice‐note‐5/file), and the Freshwater Habitats Trust's Pond Habitat Survey (https://freshwaterhabitats.org.uk/wp‐content/uploads/2015/03/HABITAT‐MANUAL‐FINAL.pdf). Further information on field assessment of the recorded environmental variables is outlined in the field data form provided. The field survey data form includes geographic coordinates, which allow important additional variables to be derived (e.g., altitude and local pond density).

For each site visit, the date/time, surveyor name, sampling location, and recorder/microphone identifiers should be recorded. A photograph of the pond can be useful (Rountree et al., 2020). Weather conditions during the survey period, especially the occurrence of rain, should also be recorded. Adverse weather should, however, generally be avoided, as this is likely to dominate the soundscape during recordings, and mask biological sounds.

## 3 APPLICATIONS FOR THE PASS

### 3.1 Survey design

Samples collected following PASS can be put to use as part of wide‐scale surveys featuring the appropriate temporal and spatial replication levels. We recommend that its use should span a range of sites and sampling periods. The phenology of different taxa through the course of a year will affect the extant assemblage in a waterbody (Aiken, 1991), and Hill et al. (2016) showed that macroinvertebrate sampling across all seasons provides the best record of the community, with autumn samples the most diverse. Gottesman et al. (2018) recommend that recordings should cover a range of seasonal and diurnal periods to capture the temporal dynamics that are part of the acoustic diversity of a given site (Decker et al., 2020; Karaconstantis et al., 2020; Kuehne et al., 2013). In addition, wide spatial coverage across numerous sites is also encouraged, as further research is needed to understand spatial heterogeneity and its effect on the variability of acoustic assessments (Linke, Gifford, et al., 2020).

### 3.2 Data storage and sharing

Several studies have highlighted the need for open science in freshwater assessments (Beck et al., 2020), and the development of open platforms to share and store freshwater recordings (Linke, Gifford, et al., 2020; Linke et al., 2018; Rountree et al., 2020). Well‐known sound archives, such as the Macaulay sound library (www.macaulaylibrary.org) and Xeno‐Canto (www.xeno‐canto.org), are mainly dedicated to bird sounds. Several other sound libraries are part of the collections of Natural History Museums such as the Sonothèque in Paris (https://sonotheque.mnhn.fr), BioAcoustica (Baker et al., 2015), or the Animal Sound Archive in Berlin (https://www.tierstimmenarchiv.de). However, most sound archives are centered on focal recordings of single species rather than location soundscapes. Moreover, in these libraries, recordings and metadata are not readily downloadable in batches for use in scientific studies.

Inspired by “Silent Cities,” a participative project to record during the COVID‐19 confinement in urban areas (https://framaforms.org/silentcities‐1584526480), we propose an integrated solution for storing and sharing recordings collected using PASS. We have set up a Zenodo community (https://zenodo.org/communities/pass) to allow the upload and validation of acoustic data and associated metadata. This dataset is freely available to anyone for scientific, educational, or artistic purposes. It is expected to provide unprecedented opportunities to unravel the potential of rapid acoustic surveys for freshwater ecological assessments.

### 3.3 Data analysis

Acoustic recordings can be analyzed in a variety of ways including manual annotation and measurements, automatic signal processing with the use of species recognizers, or integrative acoustic indices (Eldridge et al., 2018; Fuller et al., 2015; Sueur, Pavoine, et al., 2008; Wimmer et al., 2013). The PASS particularly lends itself to a rapid assessment approach using acoustic indices. The 1‐min subsamples can be processed to produce individual acoustic index scores, and these averaged to create a mean value and maximum–minimum range for the 10‐min sample. These values can then be assessed across several site visits, with metadata and environmental information being used as covariates with the analysis.

Acoustic indices are calculated by considering variations in amplitude and frequency over time in audio recordings. Their calculation can be automated and standardized, for example, using the R packages Seewave (Sueur, Aubin, et al., 2008) and Soundecology (Villanueva‐Rivera & Pijanowski, 2018), to facilitate the analysis of large data sets in a repeatable way. Gottesman et al. (2018) calculated six acoustic indices to assess the soundscape of a swamp in Costa Rica for 23 days. The study discovered clear diurnal patterns in the soundscape with active night choruses and quieter day periods.

**FIGURE 3:**
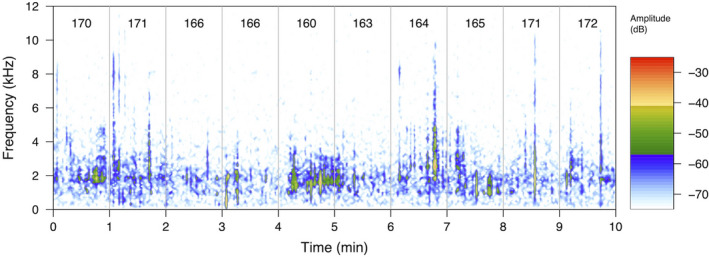
Full soundscape analysis. Spectrogram showing 10‐min sound recording, divided into 1‐min sections, each recorded in different locations around one pond. Acoustic Complexity Index (ACI) scores (range 159.9–171.8, mean 166.7) are indicated for each minute and are highest in minute 10, and lowest in minute 5. The spectrogram shows that most sound energy is centered around 1–3 kHz. Frequencies are displayed to a maximum of 12 kHz, although the recording included sounds up to 24 kHz. Spectrogram produced using package Seewave in R with an FFT size 512 and overlap = 50%. The R script for calculating the ACI scores for a recording, and producing this figure, is included in Data S1

**FIGURE 4:**
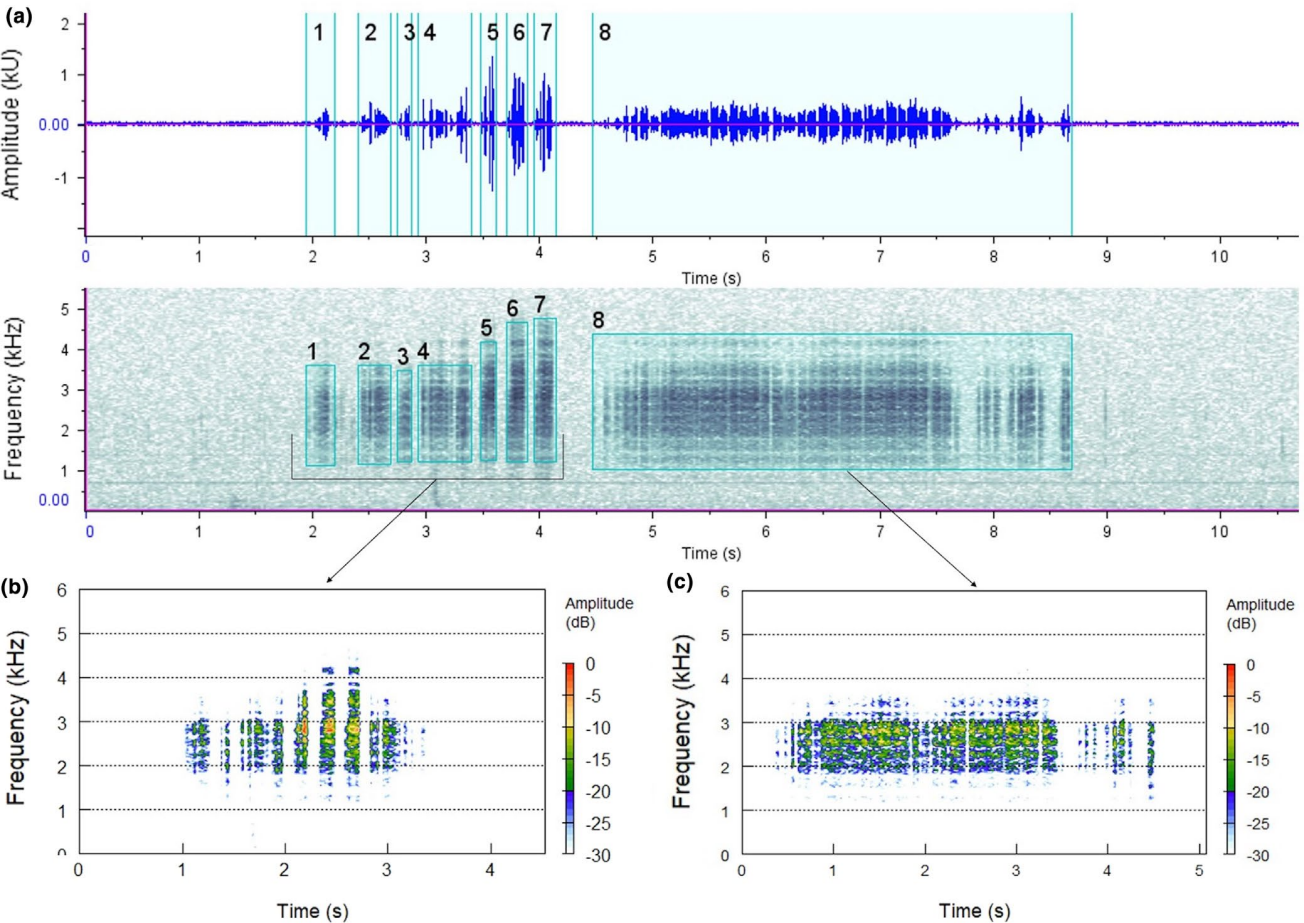
Single‐species sound analysis. Analysis of the sound types of a Corixid species: (a) waveform and spectrogram of typical Corixidae call series. Numbers 1–8 represent sections of each call series measured in Raven Pro. (b–c) Spectrograms of each sound type using the package Seewave in R with an FFT size 2,048 and overlap = 50%; (b) sound type 4, (c) sound type 8

Spectrograms visualize sound in the frequency and time domains (Figure 3) and can be generated using a variety of software to help interpret sound recordings. Some notable examples include the free and open‐source Audacity (https://www.audacityteam.org/), the R package seewave (Sueur, Aubin, et al., 2008), and Raven Pro 1.5 (https://ravensoundsoftware.com/software/raven‐pro/). These software applications also allow the user to compute a wide range of acoustic parameters, such as mean frequency or peak amplitude, which can then be exported for use in statistical analyses (Rountree & Juanes, 2020). This type of feature is demonstrated below (Figure 4), where the sounds produced by a water‐boatman have been highlighted, to allow sound parameters to be extracted and analyzed. Such signal detection and feature extraction can be done manually or automatically using signal processing such as machine learning (Browning et al., 2017).

## 4 TESTING THE PASS

During April 2020 to March 2021, we collected PASS recordings and metadata at 24 ponds across the UK. Although this was a limited pilot study, it is to our knowledge, the largest dataset yet published for pond ecoacoustics in terms of the number of sites covered. We tested the data in two ways: (1) calculating the percentage Coefficient of Variation (CV%) in an acoustic index score for the 10‐min sample and (2) comparing derived acoustic indices to the Habitat Suitability Index (HSI) for each pond.

**FIGURE 5:**
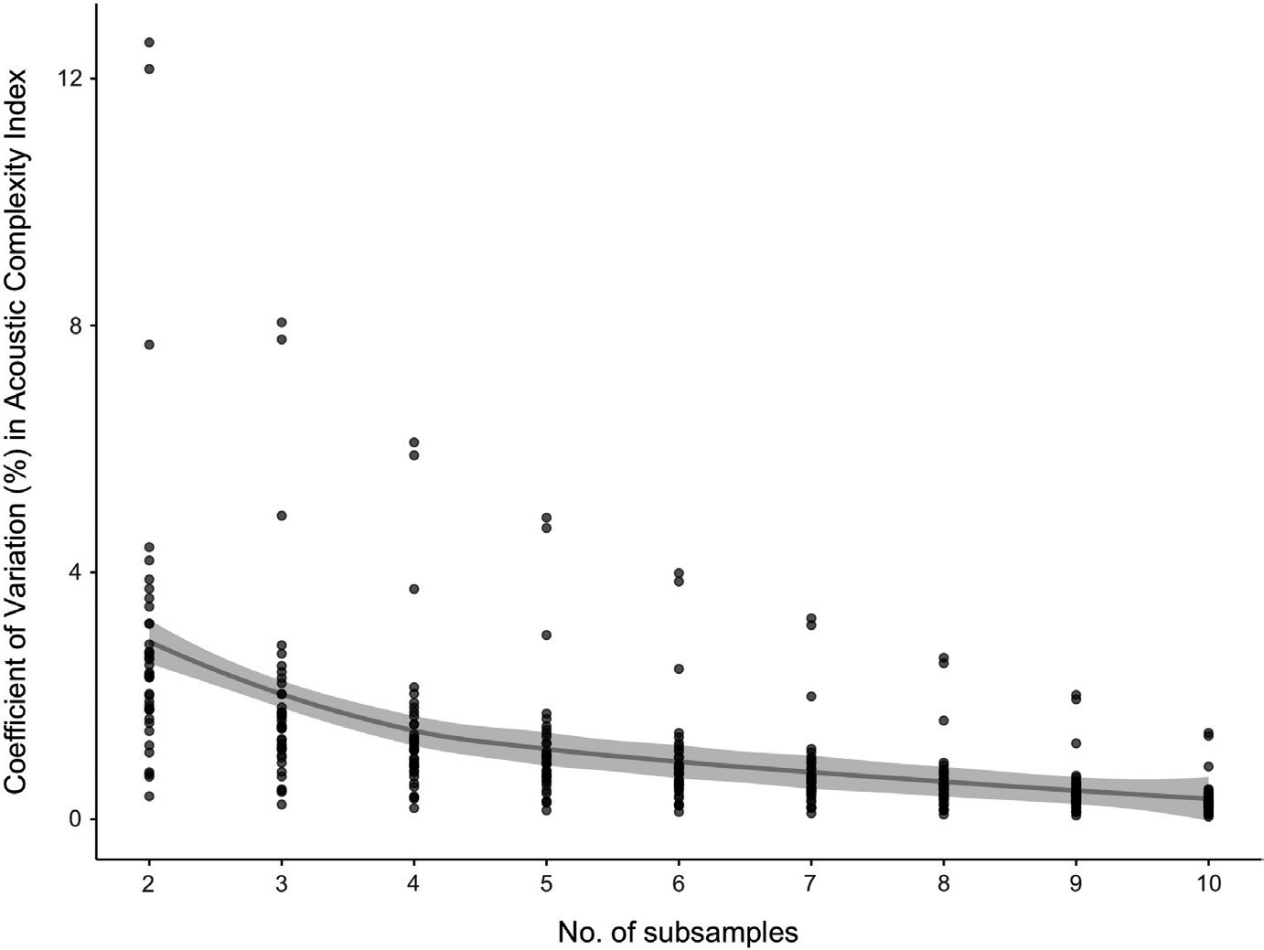
Coefficient of variation for Acoustic Complexity Index scores reduces substantially with the ten 1‐min subsamples included in the PASS protocol

Acoustic Complexity Index (ACI) scores were calculated using the seewave package in R (Sueur, Aubin, et al., 2008) for each 1‐min subsample. The CV% of the ACI score was then calculated for increasing numbers of subsamples, up to the full 10‐min recording in the sample (Figure 5). This analysis, over 33 PASS samples, shows that CV% declines substantially with ten subsamples, indicating that variation in ACI is effectively captured using the proposed recording length.

**FIGURE 6:**
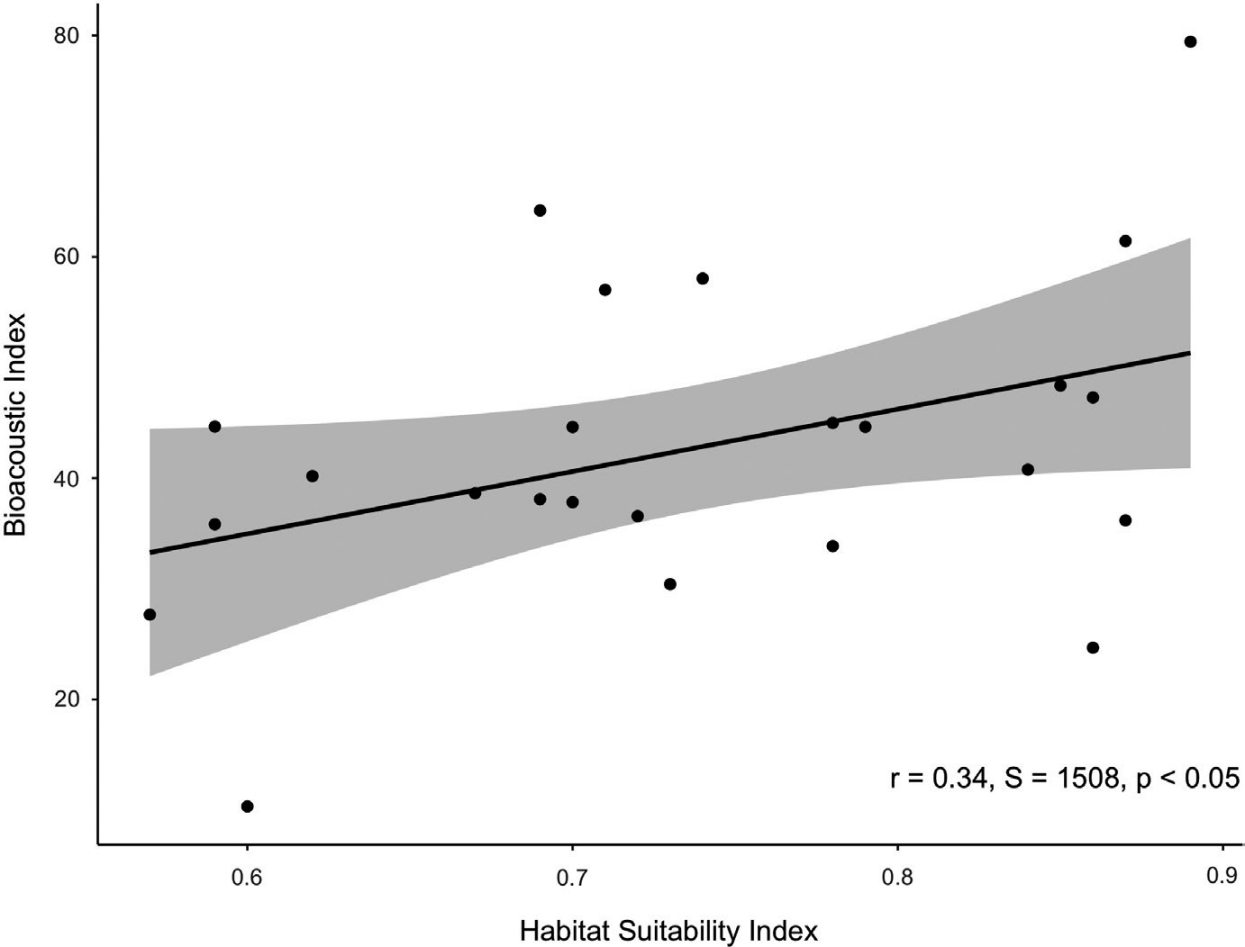
Bioacoustic Index compared with Habitat Suitability Index for 24 ponds

Environmental data collected at each PASS site was combined with a review of Ordnance Survey mapping to calculate the HSI (Oldham et al., 2000) for each pond. The HSI combines parameters such as pond area, shading, and macrophyte cover into a single value and is a well‐established metric of pond habitat quality, indicating amphibian species occupancy and abundance (Unglaub et al., 2018). A range of acoustic indices (ACI, ADI, AEI, BI, NDSI) were calculated for each site and compared with the HSI scores. Significant positive correlations were found between HSI and both ACI and the Bioacoustic Index (BI; Figure 6). This suggests that acoustic data recorded using PASS is likely to be related to a range of measurable environmental parameters and can be effectively used to assess pond habitat condition.

## 5 CONCLUSION AND OUTLOOK

The PASS offers a new and highly valuable method for consistent acoustic sampling of small waterbodies. This sampling scheme is likely to enable the rapid assessment of pond quality and condition for ecological studies and conservation management. Further development in understanding the links between the sound characteristics of ponds and their ecology is certainly needed and will require the collection and analysis of data from a large number of sites. We believe that the availability of a standard protocol for data gathering will support comparisons between studies, data sharing, and the establishment of coherent “gold‐standard” datasets. This would aid scientific research to evaluate the promising potential of ecoacoustics as a monitoring technique in small waterbodies, and better conservation action for vitally important pond habitats.

## CONFLICT OF INTEREST

None declared.

## AUTHOR CONTRIBUTION

**Carlos Abrahams:** Conceptualization (lead); Methodology (equal); Visualization (lead); Writing‐original draft (equal); Writing‐review & editing (equal). **Camille Desjonqueres:** Methodology (equal); Visualization (supporting); Writing‐original draft (equal); Writing‐review & editing (equal). **Jack Greenhalgh:** Methodology (equal); Visualization (supporting); Writing‐original draft (equal); Writing‐review & editing (equal).

## Supporting information

Data S1

## Data Availability

Audio recordings and metadata are archived at Zenodo: [https://zenodo.org/communities/pass] https://doi.org/10.5281/zenodo.3954852.
