# Supplementary material for: Pond Acoustic Sampling Scheme: A draft protocol for rapid acoustic data collection in small waterbodies
Source: Ecol Evol. 2021 May 1;11(12):7532–43. doi: 10.1002/ece3.7585 (PMC8216941; doi:10.1002/ece3.7585)
Supplement: Supplementary file 1 — Data S1 [file ECE3-11-7532-s001.zip › ece37585-sup-0001-field_data_form.pdf]

# Pond Acoustic Survey - Field Data Form

| | | | |
|---|---|---|---|
| Surveyor | | | |
| Survey Date | | Start Time (hh:mm) | |
| Pond Ref No | | Pond Name/Location | |
| Latitude (n/s) | | Longitude (w/e) | |

| | | | |
|---|---|---|---|
| Recorder Model | | Air temperature (˚C) | |
| Hydrophone Model | | Rain? (yes = 1/no = 0) | |
| Sample Rate (kHz) | | Cloud cover % | |
| Bit-depth | | Photograph references | |
| Gain setting (dB) | | | |

| | |
|---|---|
| Pond Area m² (at highest water level) | |
| Inflow (present = 1/absent = 0) | |
| Permanence (dries never, rarely, sometimes, annually) | |
| Water quality (good, moderate, poor, bad) | |
| Shading of perimeter (% of pond perimeter overhung) | |
| Macrophyte cover (% at pond surface) | |
| Waterfowl impact (absent/minor/major) | |
| Fish present? (absent, possible, minor popn, major popn) | |
| Terrestrial habitat quality around pond (good, moderate, poor, none) | |

| Audio file references | | | |
|---|---|---|---|
| 1 | | 6 | |
| 2 | | 7 | |
| 3 | | 8 | |
| 4 | | 9 | |
| 5 | | 10 | |

Notes (e.g. water depth, background noise, survey conditions)

# Pond Acoustic Survey - Field Data Form

| Optional data - water chemistry | | Water temperature (°C) | |
|---|---|---|---|
| pH | | Dissolved Oxygen (mg/L) | |
| Conductivity (μS/cm) | | Total dissolved solids (mg/L) | |

## Guidance notes for environmental variables

Pond area is the surface area of the pond when water is at its highest level (excluding flooding events). This should be evident from vegetation types and evidence of a draw down zone around the pond.

Pond permanence should be deduced from water level at the time of the survey, and taking seasonality into consideration. For example, a pond that is already dry by late spring is likely to dry out every year, etc.

- Never dries
- Rarely dries - Dries no more than two years in ten or only in drought.
- Sometimes dries - Dries between three years in ten to most years.
- Dries annually

Water quality assessment should be based on invertebrate diversity, the presence of submerged water plants and knowledge of the water sources feeding the pond. Water quality should not be confused with water clarity.

- Good - Water supports an abundant and diverse invertebrate community. Including groups such as mayfly larvae and water shrimps. Varied aquatic plant community.
- Moderate - Moderate invertebrate and plant diversity
- Poor - Low invertebrate diversity (e.g. species such as midge and mosquito larvae). Few submerged plants.
- Bad - Clearly polluted, only pollution-tolerant invertebrates (such as rat-tailed maggots), no submerged plants.

Shading of the pond perimeter should be estimated, to at least 1m from the shore. Shading is usually from trees, but can include buildings. Shading should not include emergent pond vegetation. The estimate should be made during the summer growing period.

Waterfowl impact:

- Absent - No evidence of waterfowl impact (moorhens may be present).
- Minor - Waterfowl present, but little indication of impact on pond vegetation. Pond still supports submerged plants and banks are not denuded of vegetation.
- Major - Severe impact of waterfowl. Little or no evidence of submerged plants, water turbid, pond banks showing patches where vegetation removed, evidence of provisioning waterfowl.

Terrestrial habitat should be considered within approximately 250 m from the pond:

- Good – Valuable/varied habitat composition, with semi-natural environments, such as rough grassland, scrub or woodland (or brownfield sites and low intensity farmland) over more than 75% of area.
- Moderate - Habitat offers opportunities for foraging and shelter but may not be extensive (25-75%) of available area.
- Poor - Habitat with poor structure (e.g. amenity grassland, improved pasture and arable) that offers limited opportunities (less than 25% of available area) for pond fauna.
- None - No suitable habitat around pond (e.g. centre of arable field or large expanse of bare habitat).
